# Logistic regression model for predicting risk factors and contribution of cerebral microbleeds using renal function indicators

**DOI:** 10.3389/fneur.2024.1428625

**Published:** 2024-09-18

**Authors:** Xuhui Liu, Zheng Pan, Yilan Li, Xiaoyong Huang, Xiner Zhang, Feng Xiong

**Affiliations:** ^1^Department of Neurology of the Second Hospital Affiliated to Lanzhou University, Lanzhou, China; ^2^Jinshan Branch of Shanghai Sixth People’s Hospital, Shanghai, China; ^3^Tianjin 4th Center Hospital, Tianjin, China; ^4^Department of Cardiology, Lishui People’s Hospital, The Sixth Affiliated Hospital of Wenzhou Medical University, Lishui, China; ^5^Department of Medical Oncology, Affiliated Tumor Hospital of Xinjiang Medical University, Ürümqi, China

**Keywords:** cerebral microbleeds, renal function indicators, hypertension, risk factors, contribution

## Abstract

**Background:**

The brain and kidneys share similar low-resistance microvascular structures, receiving blood at consistently high flow rates and thus, are vulnerable to blood pressure fluctuations. This study investigates the causative factors of cerebral microbleeds (CMBs), aiming to quantify the contribution of each risk factor by constructing a multivariate model via stepwise regression.

**Methods:**

A total of 164 hospitalized patients were enrolled from January 2022 to March 2023 in this study, employing magnetic susceptibility-weighted imaging (SWI) to assess the presence of CMBs. The presence of CMBs in patients was determined by SWI, and history, renal function related to CMBs were analyzed.

**Results:**

Out of 164 participants in the safety analysis, 36 (21.96%) exhibited CMBs and 128 (78.04%) did not exhibit CMBs, and the median age of the patients was 66 years (range: 49–86 years). Multivariate logistic regression identified hypertension (OR = 13.95%, 95% CI: 4.52, 50.07%), blood urea nitrogen (BUN) (OR = 1.57, 95% CI: 1.06–2.40), cystatin C (CyC) (OR = 4.90, 95% CI: 1.20–22.16), and urinary β-2 microglobulin, (OR = 2.11, 95% CI: 1.45–3.49) as significant risk factors for CMBs. The marginal *R*-square ( $R_{M}^{2}$ ) was 0.25. Among all determinants, hypertension (47.81%) had the highest weight, followed by UN (11.42%). Quasi-curves plotted using the bootstrap method (999 times) showed good agreement between the predictive model and actual observations.

**Conclusion:**

Hypertension, BUN, urinary β-2 microglobulin, CyC were risk factors for CMBs morbidity, and controlling the above indicators within a reasonable range will help to reduce the incidence of CMBs.

## 1 Introduction

Cerebrovascular disease encompasses a broad spectrum of conditions that impair the normal function of cerebral blood vessels, significantly raising the risk of severe outcomes such as ischemic and hemorrhagic strokes (1). Recently, there has been growing interest in cerebrovascular diseases, specifically cerebral microbleeds (CMBs), due to their association with many diseases.

CMBs have distinct pathological and imaging characteristics that are crucial for their diagnosis and understanding. CMBs are characterized by small hemorrhages around cerebral blood vessels, marked by the presence of macrophages containing hemosiderin (2). These lesions appear as small, round areas with low signal intensity on magnetic resonance imaging (MRI) using gradient recall echo sequences (3). While CMBs can be observed in healthy older individuals, they can also serve as predictive markers for future stroke, particularly hemorrhagic stroke, in patients receiving antithrombotic medication (4). Different underlying causes contribute to the development of CMBs, including amyloid angiopathy and hypertension, each with distinct distribution patterns (5). Amyloid angiopathy-related CMBs are typically found in lobular brain structures, while hypertension-induced CMBs tend to occur in deep brain tissues like the basal ganglia, thalamus, cerebellum, and brainstem (6). Managing and preventing CMBs necessitates considering the interplay between hypertension and renal function impairment as important factors. Both the kidneys and the brain possess similar low-resistance microvascular structures that receive blood at sustained high flow rates (7). Consequently, impairments in renal function may contribute to pathological changes in small and medium cerebral vessels, particularly the terminal arteries (8). Although previous studies have explored markers for subclinical kidney and brain diseases, few have comprehensively examined how these markers correlate with damage to both organs.

Hence, this study evaluated the clinical characteristics and risk factors related to renal function in patients with the onset of CMBs, and constructed a multivariate logistic model combining the risk factors with a view to providing a reference for clinical diagnosis.

## 2 Materials and methods

### 2.1 Patients

**Figure 1:**
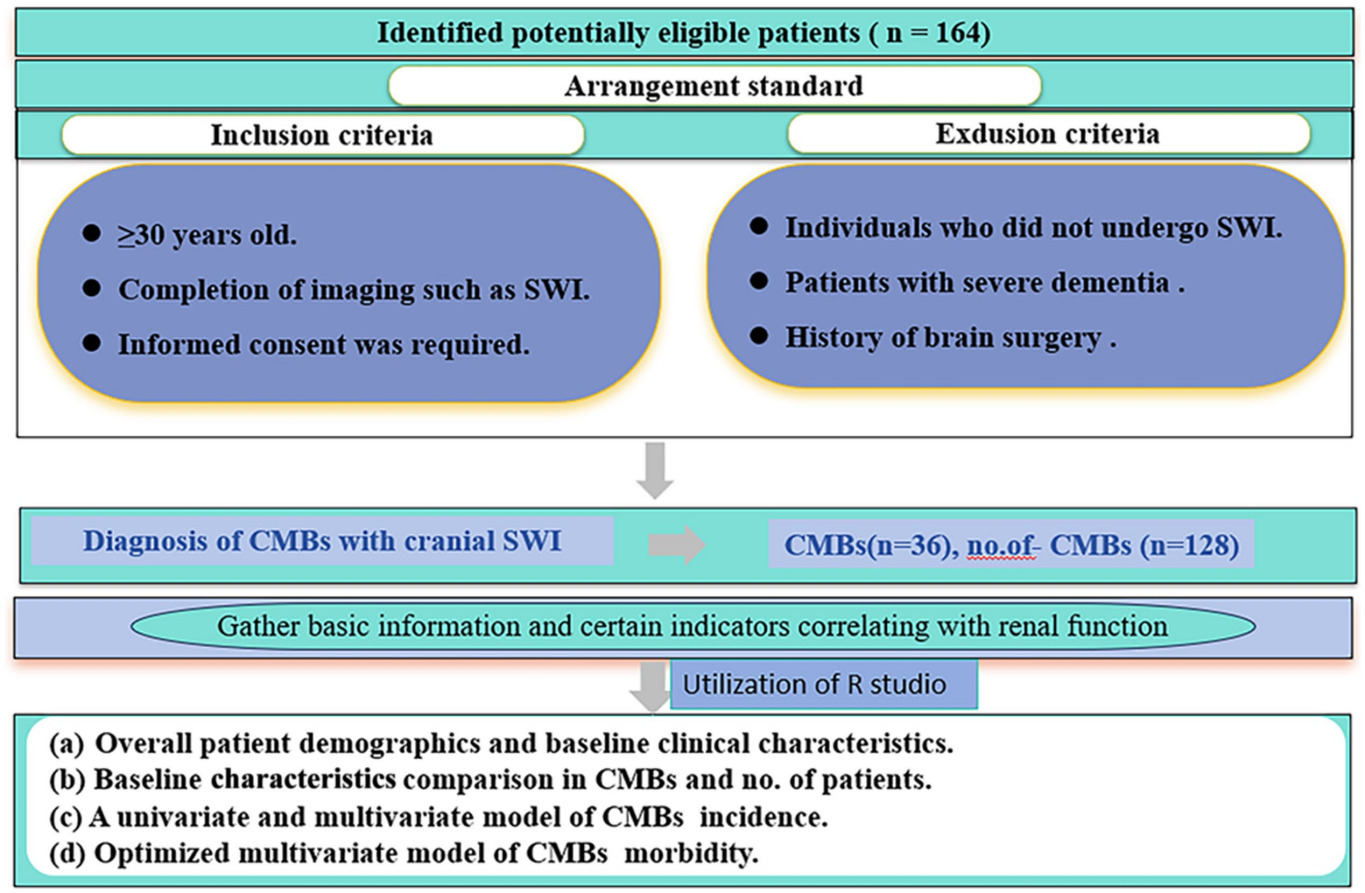
Flow chart of patient selection and study design.

Between January 2022 and March 2023, a retrospective study was conducted in the Department of Neurology at the Second Hospital of Lanzhou University. The aim of the study was to examine patients aged 50 years or older who sought medical attention for various neurologic symptoms. The inclusion criteria were as follows (i) ≥30 years of age; (ii) completion of imaging; and (iii) signing of informed consent. Exclusion criteria were as follows (i) patients with severe dementia; (ii) patients with a history of brain surgery; and (iii) patients who did not undergo magnetic resonance imaging. Patient demographics (gender, age, smoking, alcohol consumption), underlying diseases (hypertension, diabetes mellitus), laboratory tests (renal function, blood glucose, glycated hemoglobin) and other relevant information were obtained from the hospital’s electronic medical record system. Informed consent was obtained from all patients included in the study, and they subsequently underwent magnetic induction-weighted imaging (SWI) of the head. The study protocol was approved by the Medical Ethics Committee of the Second Hospital of Lanzhou University, China). The flow chart of this study is shown in Figure 1.

### 2.2 Definition of patients and CMBs

After completion of all MRI scans, diagnoses were made by MRI by two neurologists with extensive clinical experience, and participants were divided into two groups: a CMBs group and a non-CMBs control group. MRI scans were performed using a 1.5 Tesla scanner from General Electric Healthcare (Signa, Milwaukee, Wisconsin) or Siemens (Magnetom; SONATA, Munich, Germany). The imaging program consisted of the following sequences: a T2-weighted fast spin echo (TR/TE = 5000/127 ms), a T1-weighted spin echo (TR/TE = 500/11 ms), and fluid-attenuated inversion recovery (TR/TE = 8,800/127 ms; inversion time = 2,250 ms). Each sequence consists of 26 axial slices with a slice thickness of 5 mm and a slice spacing of 1 mm.

CMBs were characterized with well-defined boundaries showing low signal intensity, accompanied by halo stains, and having a diameter smaller than 5 mm (9). Two attending physicians or higher qualified doctors from the Department of Neurology independently reviewed the computerized MR images of each patient. In the case of disagreements, a consensus was reached through discussion with a third reviewer (10).

In this study, the diagnosis of diabetes was confirmed based on either the administration of antidiabetic treatment or repeated pathological blood tests indicating a fasting glucose level equal to or exceeding 7 mmol/L (126 mg/dL) or a postprandial glucose level equal to or surpassing 11.1 mmol/L (200 mg/dL) 2 h after oral glucose intake. Conversely, hypertension was defined as having a blood pressure measurement equal to or higher than 140/90 mmHg or receiving antihypertensive medication during three distinct time points.

### 2.3 Measurement of renal function indicators

After an overnight fasting period, blood samples were collected from the patients typically between 6:00 and 8:00 in the morning. Standard laboratory techniques were utilized to measure the levels of urea nitrogen, uric acid, urine microalbumin, urinary α1 microglobulin, and urinary β-2 microglobulin. These measurements were performed using a Hitachi 7600 automated analyzer, with high-sensitivity C-reactive protein (hs-CRP) levels analyzed through latex-enhanced immune turbidimetry (Hitachi, Tokyo, Japan). Serum CyC levels were determined using the same analyzer but employing automatic particle-enhanced immune turbidimetry. Serum creatinine levels were measured using the Jaffe kinetic method computerized analyzer.

### 2.4 Statistical analysis

Continuous variables are expressed as mean ± standard deviation (mean ± SD) or median (IQR), and categorical variables as percentages [*n* (%)]. Normality was assessed using *t*-test or Mann–Whitney *U*-test for continuous variables, while chi-square test or two-tailed Fisher’s exact test were used for categorical variables. The strength of correlation was assessed by odds ratio (OR) and 95% confidence interval (CI). Univariate logistic regression analyses were performed, followed by multivariate analyses for variables with *p*-values <0.05 to identify CMBs factors. Model discriminability was validated using 999 bootstrap sample calibration plots. The relative contribution of determinants to CSVD incidence was assessed by calculating partial *R*-squared (*R*^2^) and contribution percentages. For hypertension severity analysis, patients were categorized based on systolic blood pressure (<120 mmHg, 120–139 mmHg, ≥140 mmHg), with CMBs incidence compared using chi-square tests. ROC curve analysis determined the optimal cutoff value for urinary β-2 microglobulin in predicting CMBs, calculating AUC, sensitivity, and specificity. Subgroup analysis compared CMBs risk factors between diabetic and non-diabetic patients, using two-way ANOVA to assess interactions. A comprehensive renal function scoring system was developed using nomogram analysis, integrating CyC, blood urea nitrogen (BUN), urinary β-2 microglobulin, and hypertension status to analyze CMBs risk. Statistical significance was set at *p* < 0.05, with all analyses performed in R (version 4.2.3).

## 3 Results

### 3.1 Overall patient demographics and baseline clinical characteristics

Overall, 164 patients were enrolled in the safety analysis set (Figure 1). The median age of patients was 66 years (range: 49–86 years), 36 cases in CMBs, 128 cases in no. of patients, and the other characteristics of patients were shown in Table 1.

**Table 1:** Demographics and baseline clinical characteristics of patients.

Variable		Safety analysis set (*n* = 164)
Sex		
	Female	82 (50%)
	Male	82 (50%)
Hypertension		
	No	111 (67.68%)
	Yes	53 (32.32%)
Diabetes		
	No	129 (78.66%)
	Yes	35 (21.34%)
Smoking		
	No	142 (86.59%)
	Yes	22 (13.41%)
Drinking		
	No	149 (90.85%)
	Yes	15 (9.15%)
Cerebral microbleeds (CMBs)		
	No	128 (78.04%)
	Yes	36 (21.96%)
Age (years)		65.54 ± 8.11
Body mass index (BMI, kg/m^2^)		24.47 ± 3.01
Systolic blood pressure (SBP, mmHg)		132.68 ± 14.69
Fasting glucose (FG, mmol/L)		5.42 ± 1.38
Glycated hemoglobin (HbA1c, mmol/L)		5.97 ± 1.15
Urea nitrogen (BUN, mmol/L)		5.75 ± 1.59
Uric acid (UA, μmol/L)		306.09 ± 90.27
Creatinine (Cr, μmol/L)		82.30 ± 34.34
Cystatin C (CyC, mg/L)		1.06 ± 0.04
Systolic pressure (mmHg)		132.68 ± 14.69
C-reactive protein (CRP, mmol/L)		1.39 ± 1.79
Microalbuminuria (mg/L)		9.40 ± 16.94
Urinary α-1 microglobulin (mg/L)		9.36 ± 10.60
Urinary β-2 microglobulin (mg/L)		0.90 ± 1.18

### 3.2 Baseline characteristics comparison in CMBs and no. of patients

The baseline characteristics and number of patients with CMBs are presented in Table 2. There was no difference in the age of patients with CMBs (65.60 ± 9.20) years and no. of patients (66.50 ± 7.80) between the two groups. Variables such as gender, age, smoking, drinking, BMI, gender, FG, HbA1c, CRP, and Cr did not differ between the two groups. Hypertension, diabetes mellitus, SBP, UN, UA, CyC, microalbuminuria, urinary α1 microglobulin, and urinary β-2 microglobulin differed significantly between the number of CMBs and no of patients. The UN, UA, and CyC of patients with CMBs, microalbuminuria, urinary α1 microglobulin, urinary β-2 microglobulin were significantly higher in patients with CMBs than in non-patients.

**Table 2:** Comparison of demographics and baseline clinical characteristics in CMBs and no. of patients.

Variable	Levels	CMBs (*N* = 128)	Non-CMBs (*N* = 36)	*p*
Age		65.6 ± 9.2	66.50 ± 7.80	0.991
Smoking	No	114 (89.1%)	28 (77.8%)	0.139
Drinking	No	119 (93%)	30 (83.3%)	0.149
	Yes	9 (7%)	6 (16.7%)	
Gender	Female	63 (49.2%)	19 (52.8%)	0.850
	Male	65 (50.8%)	17 (47.2%)	
Hypertension	No	103 (80.5%)	8 (22.2%)	<0.001
	Yes	25 (19.5%)	28 (77.8%)	
Diabetes	No	106 (82.8%)	23 (63.9%)	0.027
	Yes	22 (17.2%)	13 (36.1%)	
SBP (mmHg)		131.3 ± 15.0	137.6 ± 12.3	0.023
BMI (kg/m^2^)		24.3 ± 2.9	25.0 ± 3.3	0.237
Age (years)		65.5 ± 7.8	65.6 ± 9.2	0.991
FG (mmol/L)		5.4 ± 1.4	5.5 ± 1.2	0.745
HbA1c, (mmol/L)		5.9 ± 1.1	6.2 ± 1.4	0.252
BUN (mmol/L)		5.4 ± 1.4	7.0 ± 1.6	<0.001
UA (μmol/L)		295.6 ± 88.0	343.5 ± 89.4	0.005
Cr (μmol/L)		80.3 ± 36.0	89.5 ± 26.8	0.095
CRP (mmol/L)		1.4 ± 1.9	1.5 ± 1.4	0.822
CyC (mg/L)		1.0 ± 0.3	1.3 ± 0.5	<0.001
Microalbuminuria (mg/L)		7.3 ± 16.9	16.8 ± 15.1	0.003
Urinary α-1 microglobulin (mg/L)		8.5 ± 10.0	12.4 ± 12.2	0.048
Urinary β-2 microglobulin (mg/L)		0.7 ± 0.9	1.7 ± 1.6	<0.001

**Figure 2:**
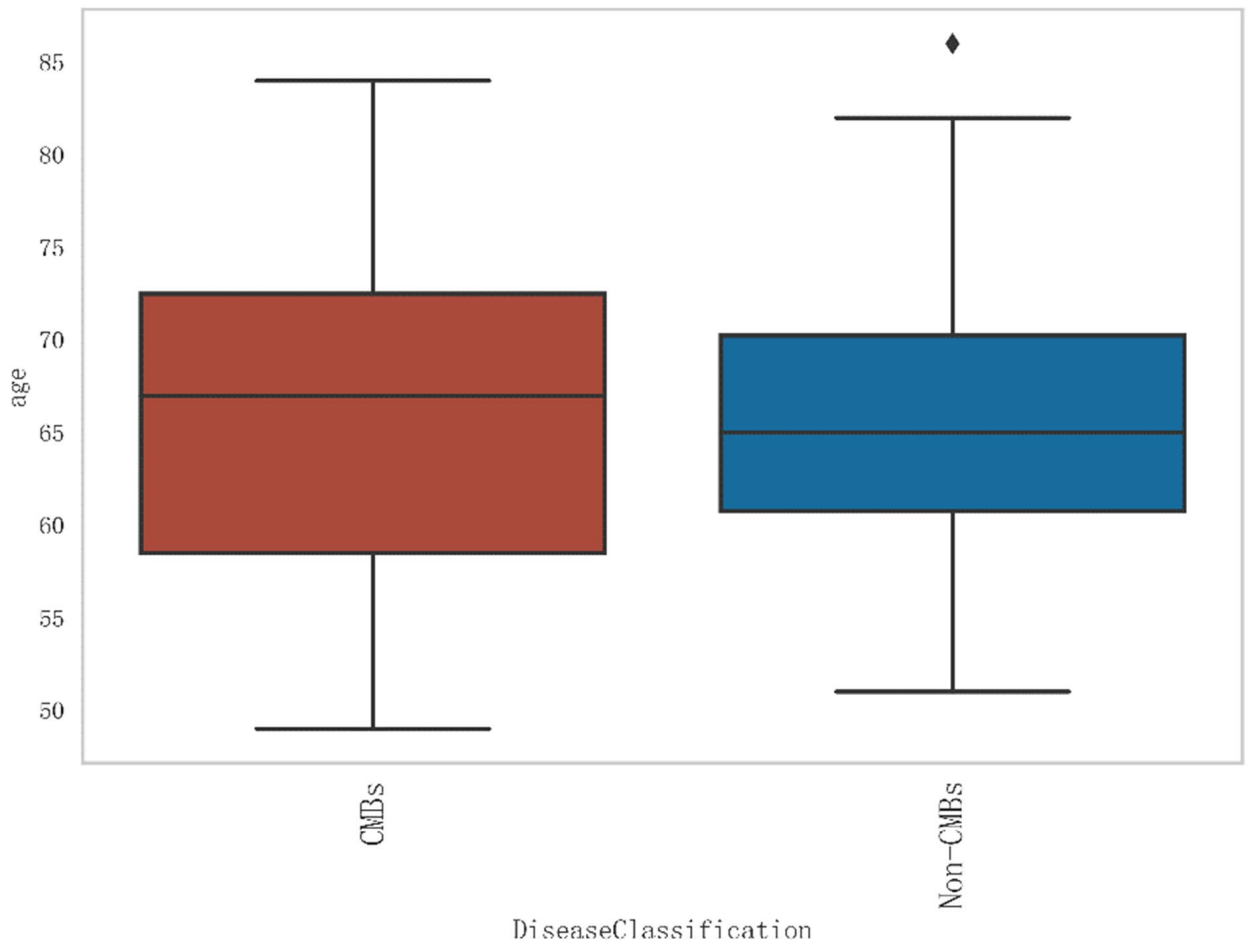
Age distribution of patients with and without CMBs.

**Figure 3:**
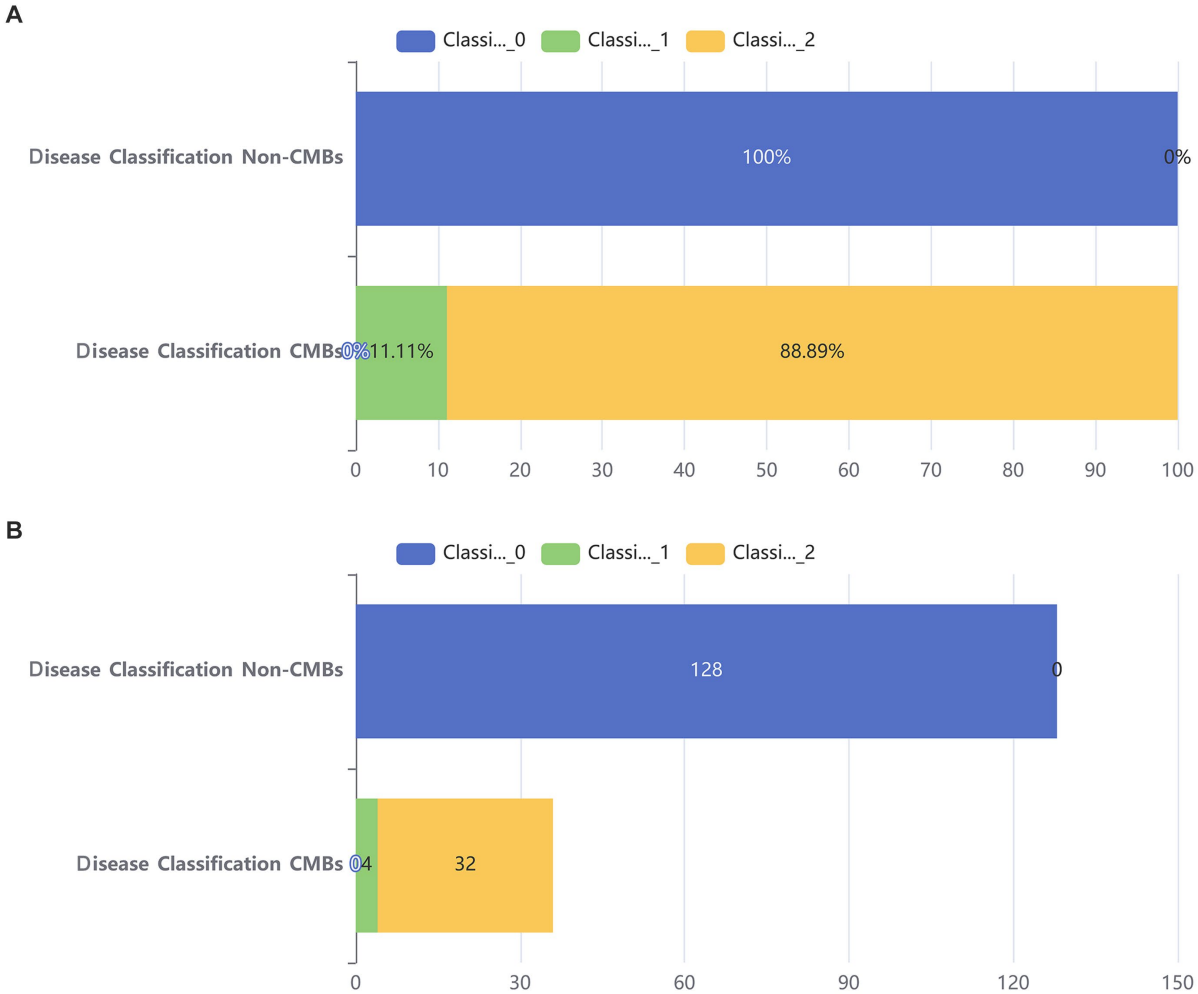
Bar chart of two groups. **(A)** Stacked bar chart of percentages between two groups. **(B)** Counting stacked bar chart in two groups of population.

Additionally, from Figure 2, it can be seen that the median age of the CMBs group is 68 years, while the median age of the non-CMBs group is 65 years. The median age of the CMBs group is slightly higher than that of the non-CMBs group. The age distribution range of the CMBs group is 50 to 85 years old, while the age distribution range of the non-CMBs group is 50 to 80 years old, and the age distribution of the CMBs group is more extensive. Although the median age difference between the two groups is not significant, the interquartile range (IQR) of the CMBs group is 15 years (from 60 to 75 years), and the interquartile range (IQR) of the non-CMBs group is 10 years (from 60 to 70 years), indicating a larger age range and interquartile range for the CMBs group. As shown in Figure 3 the majority of CMB’s were in the deep brain territory suggesting a more likely hypertensive contribution than CAA. It can be seen that in the CMBs group, 4 patients belong to the non-basal ganglia area of cerebral hemorrhage, accounting for 11.11%, and 32 patients belong to the basal ganglia area of cerebral hemorrhage, accounting for 88.89%. In the non-CMBs group, all 128 patients had no CMBs, accounting for 100%. This indicates a significant difference in disease classification between patients with cerebral hemorrhage and those without cerebral hemorrhage. Classification 0 indicates no occurrence of CMBs, classification 1 indicates that CMBs occur in non-basal ganglia areas, and classification 1 indicates that CMBs occur in basal ganglia areas.

### 3.3 Collinearity analysis

The result of collinearity analysis conducted among the variables related to CMBs, using the variance inflation factor (VIF) to assess the degree of correlation between the variables (Table 3). A higher VIF value indicates stronger collinearity between the variables. The VIF values for most of the variables observed in the table are between 1 and 2, suggesting relatively low collinearity between them, and thus a minimal impact on the model. For instance, the variables such as urinary α1 microglobulin, urea nitrogen, fasting blood glucose, glycosylated hemoglobin, urinary β-2 microglobulin, and CyC, with VIF values around 1.5, provide relatively independent information, contributing to the study of factors related to CMBs. Overall, most variables exhibit low collinearity. Consequently, the regression models built upon these variables are typically more stable in their coefficient estimation. The smaller standard errors in parameter estimation help improve the accuracy, stability, and interpretability of regression analysis, enabling the model to more effectively describe and predict changes in the dependent variable.

**Table 3:** The results of collinearity analysis.

Variable	VIF
Urinary α-1 microglobulin (mg/L)	1.734
Urea nitrogen (BUN, mmol/L)	1.732
Fasting glucose (FG, mmol/L)	1.718
Glycated hemoglobin (HbA1c, mmol/L)	1.682
Urinary β-2 microglobulin (mg/L)	1.618
Cystatin C (CyC, mg/L)	1.555
Hypertension	1.493
Diabetes	1.485
Microalbuminuria (mg/L)	1.382
C-reactive protein (CRP, mmol/L)	1.291
Smoking	1.271
Uric acid (UA, μmol/L)	1.265
Drinking	1.236
Creatinine (Cr, μmol/L)	1.235
Systolic blood pressure (SBP, mmHg)	1.229
Sex	1.197
Age (years)	1.191
Body mass index (BMI, kg/m^2^)	1.097

### 3.4 Univariate and multivariate modeling of the incidence of CMBs

As shown in Table 4, univariate regressions were performed on the variables for which there was a significant difference between CMBs and the number of patients. Table 5 summarizes the results of the multivariate logistic model, in which the significant determinants are described. The results showed that Hypertension, BUN, CyC, and urinary β-2 microglobulin were significant determinants among all factors. People with hypertension had 13.95% (95% CI, 4.52–50.07) compared to those without hypertension. For every 1 mmol/L increase in BUN, the incidence of CMBs increased by 1.57% (95% CI, 1.06–2.40). For every 1 mmol/L increase in CyC, the incidence of CMBs increased by 4.90% [95% CI: for each 1 mmol/L increase in CyC, there was a 4.90% (95% CI, 1.20–22.16) increase in the incidence of CMBs]. For each 1 mmol/L increase in urinary β-2 microglobulin, the incidence of CMBs increased by 2.11% (95% CI, 1.45–3.49).

**Table 4:** Results from the optimized multivariate model of CMBs morbidity.

Variable		Non-CMBs (*n* = 128)	CMBs (*n* = 36)	OR 95% CI	*p*-value
Hypertension					
	No	103 (80.47%)	8 (22.22%)	Reference	
	Yes	25 (19.53%)	28 (77.78%)	13.9 (5.88; 36.6)	<0.001
Diabetes					
	No	106 (82.81%)	23 (63.89%)	Reference	
	Yes	22 (17.19%)	13 (36.11%)	2.71 (1.17; 6.18)	0.021
SBP (mmHg)		131.30 ± 15.05	137.58 ± 12.32	1.03 (1.00; 1.06)	0.026
BUN (mmol/L)		5.39 ± 1.39	7.02 ± 1.64	2.71 (1.17; 6.18)	<0.001
UA (μmol/L)		295.56 ± 88.02	343.50 ± 89.42	1.01 (1.00; 1.01)	0.006
CyC (mg/L)		0.99 ± 0.32	1.33 ± 0.53	7.84 (2.98; 20.7)	<0.001
Urinary β-2 microglobulin (mg/L)		0.69 ± 0.94	1.68 ± 15.07	2.45 (1.33; 4.51)	0.004
Microalbuminuria (mg/L)		7.33 ± 16.91	16.76 ± 1.39	1.04 (1.01; 1.07)	0.022

**Table 5:** Results from the multivariate model of CMBs morbidity.

Variable		*n*	OR 95% CI	*p*-value
Hypertension				
	No	111	Reference	
	Yes	53	13.95 (4.52, 50.07)	<0.001
Diabetes				
	No	129	Reference	
	Yes	35	1.93 (0.58, 6.34)	0.28
SBP (mmHg)		164	0.98 (0.94, 1.02)	0.42
BUN (mmol/L)		164	1.57 (1.06, 2.40)	0.03
UA (μmol/L)		164	1.00 (1.00, 1.01)	0.39
CyC (mg/L)		164	4.90 (1.20, 22.16)	0.03
Microalbuminuria (mg/L)		164	1.00 (0.97, 1.03)	0.96
Urinary β-2 microglobulin (mg/L)		164	2.11 (1.45, 3.49)	<0.001

### 3.5 Receiver operating characteristic curve analysis

**Figure 4:**
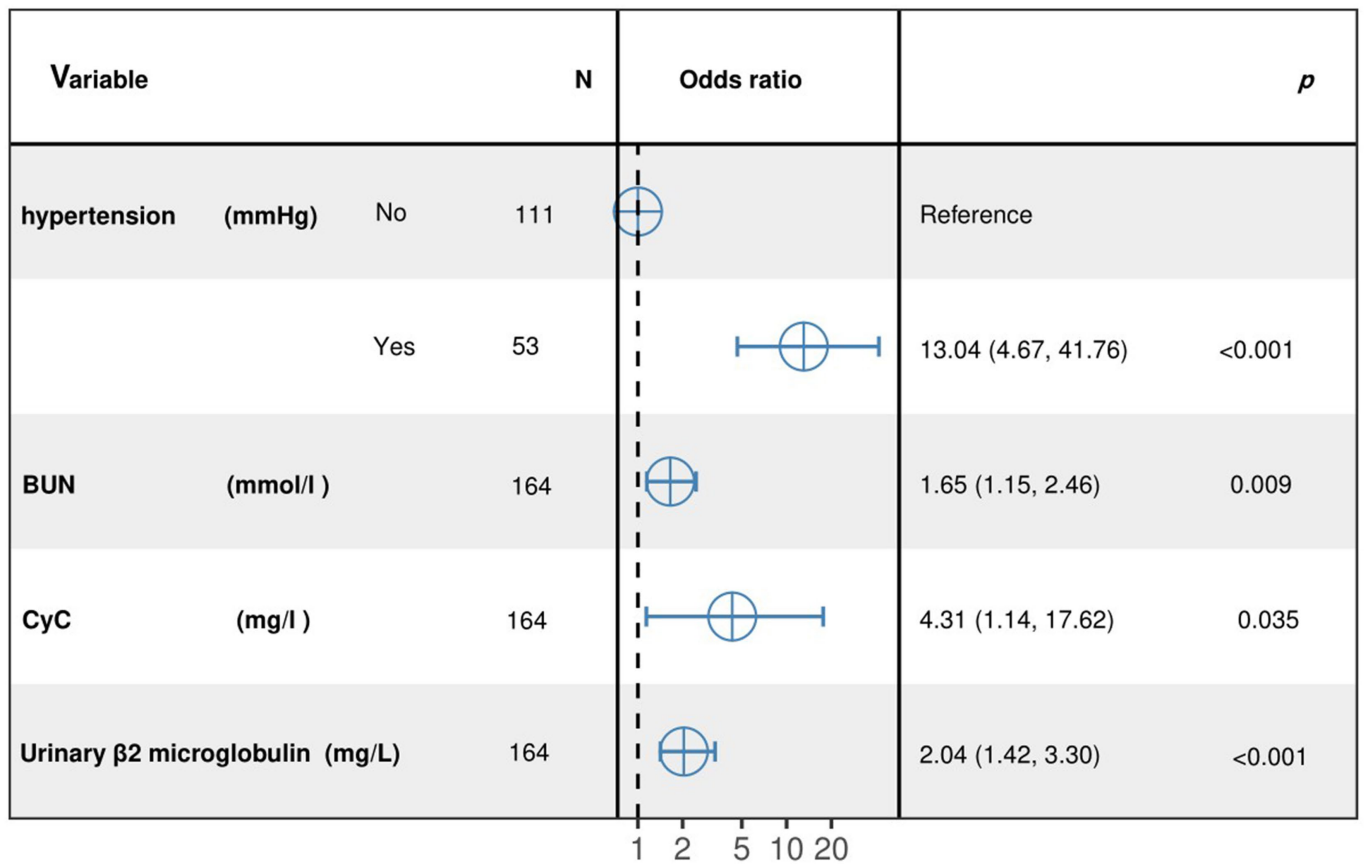
Results from the optimized multivariate model of CMBs morbidity.

**Figure 5:**
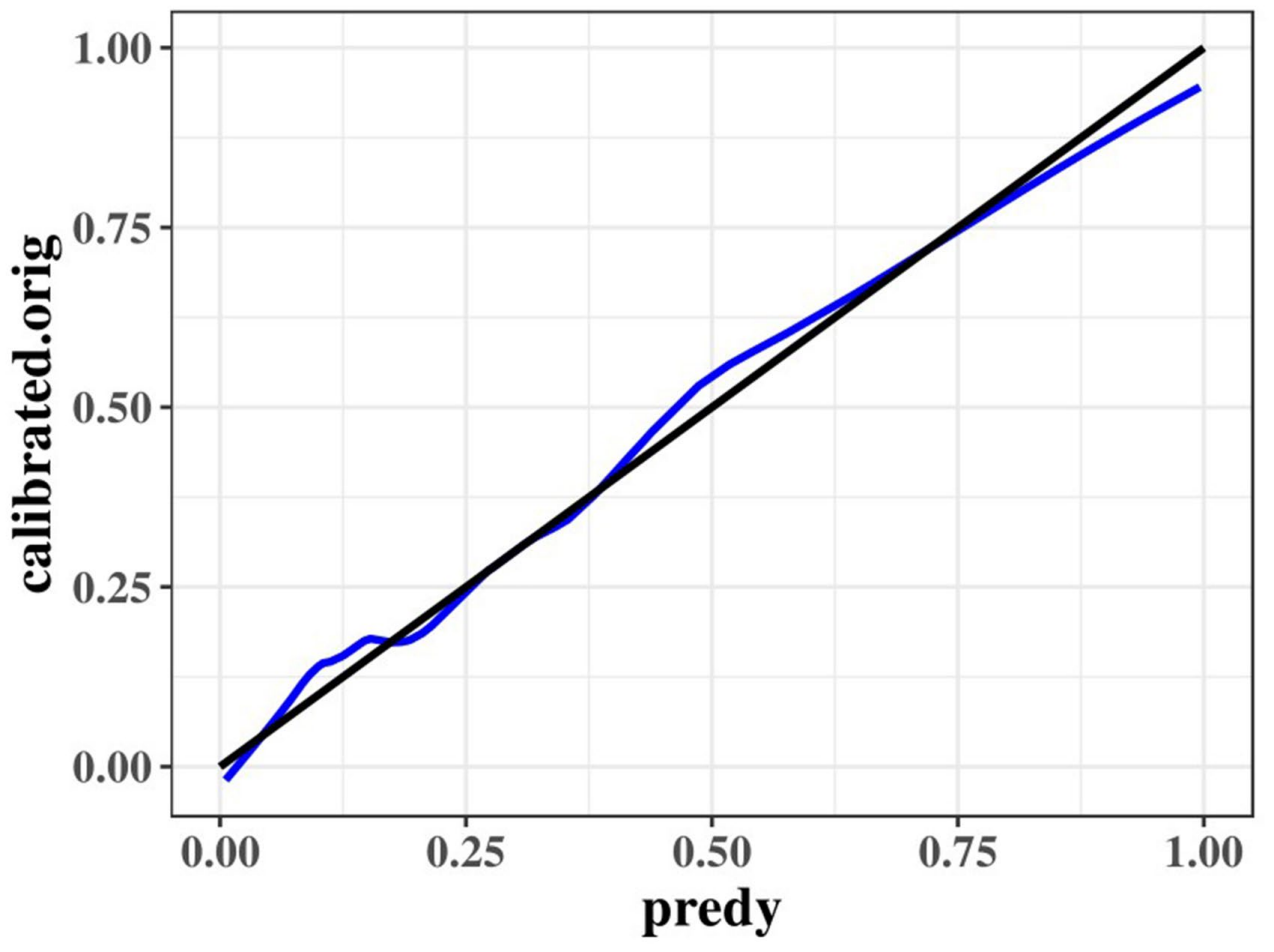
Calibration curves of the optimized multivariate model for predicting predict the probability of CMBs.

The results of the optimized multivariate model are shown in Figure 4. Compared with those without hypertension levels, 13.04% (95% CI: 4.67–41.76, *p* < 0.001) had hypertension levels. Each 1 mmol/L increase in BUN was associated with a 1.65% (95% CI: 1.15–2.46, *p* = 0. 009) increase in the incidence of CMBs. Each 1 mmol/L increase in CyC was associated with a 4.31% (95% CI, 1.14–17.62, *p* = 0.00) increase in the incidence of CMBs. The results of the optimized multivariate model were shown in Figure 4 incidence increased by 4.31% (95% CI, 1.14–17.62, *p* = 0.04) for every 1 mmol/L increase in CyC, there was a 2.04% (95% CI, 1.42–3.30, *p* < 0.01) increase in the incidence of CMBs. Table 6 shows the results of the final multivariate modeling by stepwise regression, which illustrates the contribution of several variables. The marginal *R*-squared ( $R_{M}^{2}$ ) was 0.245. Among all determinants, Hypertension concentration had the greatest effect on CSVD incidence with 47.81%, followed by BUN (11.42%), urinary β-2 microglobulin (6.93%), and CyC (4.49%). To prevent bias in the results, a constructed calibration curve was used in this study (Figure 5). The calibration curves plotted using the bootstrap method (999 times) showed good agreement between the predictive model and the actual observations.

**Table 6:** The partial *R*-squared ( $R_{\beta }^{2}$ ) of the optimized multivariate model.

Variable		$R_{\beta }^{2}$	Contribution (%)
Hypertension			
	No	Reference	—
	Yes	0.117	47.81%
BUN (mmol/L)		0.028	11.42%
Urinary β-2 microglobulin (mg/L)		0.017	6.93%
CyC (mg/L)		0.011	4.49%

### 3.6 A logistic regression model for predicting risk factors and contributions to cerebral microhemorrhage based on renal function indicators

#### 3.6.1 Stratified analysis of systolic blood pressure and CMBs risk

**Figure 6:**
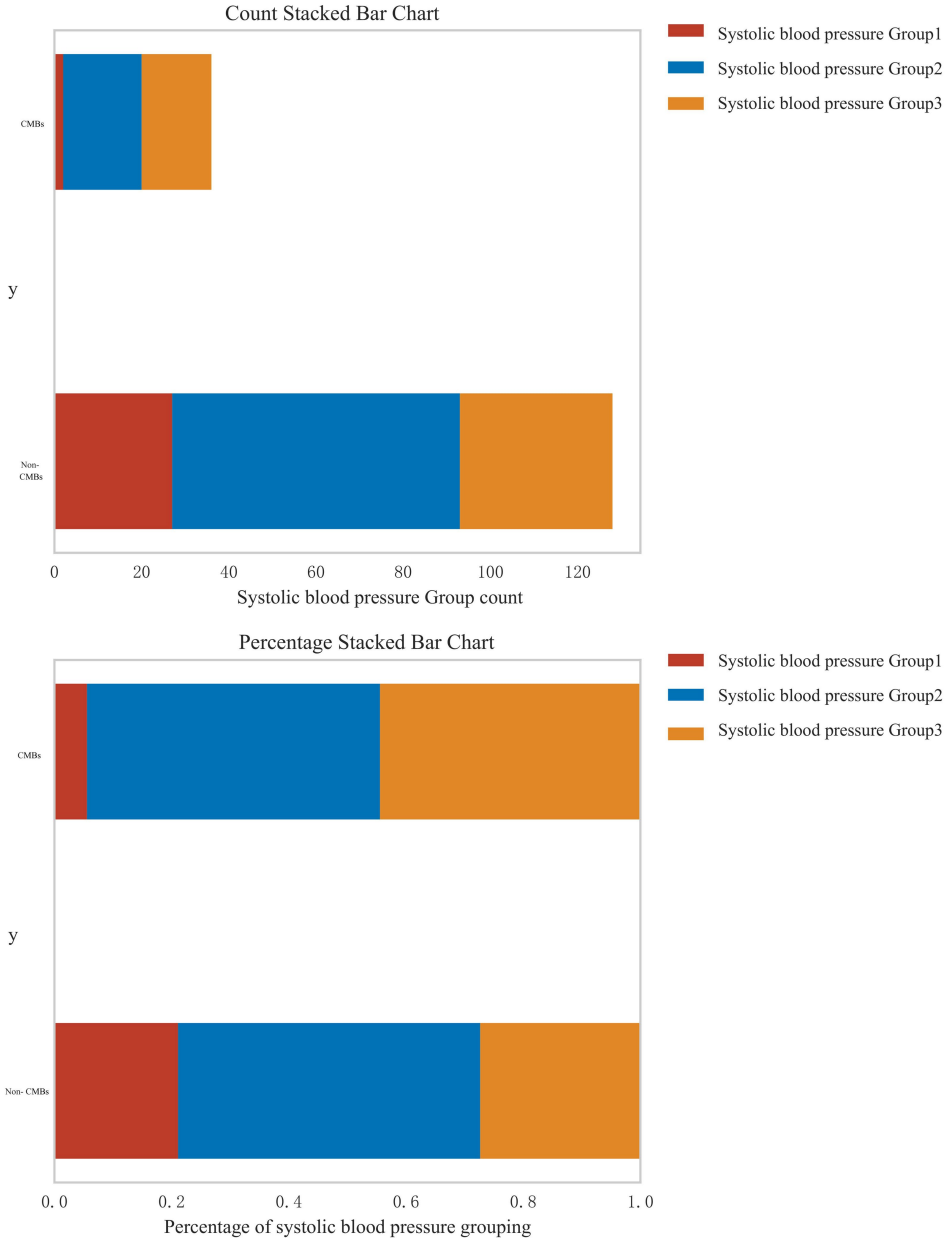
Distribution of CMBs across systolic blood pressure categories.

In response to the reviewer’s suggestion, we conducted a stratified analysis to examine the relationship between systolic blood pressure (SBP) levels and the risk of CMBs. Patients were categorized into three groups based on their SBP: normal blood pressure (<120 mmHg), pre-hypertension (120–139 mmHg), and hypertension (≥140 mmHg). Figure 6 presents the distribution of CMBs across these blood pressure categories using both count and percentage stacked bar charts. The analysis revealed a clear association between increasing SBP levels and the prevalence of CMBs. In the CMBs group, the majority of patients fell into the highest SBP category (Group 3, ≥140 mmHg), accounting for approximately 60% of CMBs cases. The intermediate SBP group (Group 2, 120–139 mmHg) represented about 30% of CMBs cases, while the lowest SBP group (Group 1, <120 mmHg) accounted for roughly 10% of CMBs cases. Conversely, in the non-CMBs group, the distribution across SBP categories was more evenly spread. Group 1 (<120 mmHg) accounted for approximately 35% of non-CMBs cases, Group 2 (120–139 mmHg) for about 40%, and Group 3 (≥140 mmHg) for roughly 25%. The percentage stacked bar chart further emphasizes this trend. Among patients with CMBs, there is a clear predominance of those with higher SBP levels, whereas the non-CMBs group shows a more balanced distribution across SBP categories. This stratified analysis strongly suggests a dose-response relationship between systolic blood pressure levels and the risk of CMBs. The proportion of patients with CMBs progressively increases from the normal blood pressure group to the hypertension group, underscoring the potential role of elevated blood pressure as a significant risk factor for CMBs development.

#### 3.6.2 Urinary β-2 microglobulin as a predictor of CMBs

**Figure 7:**
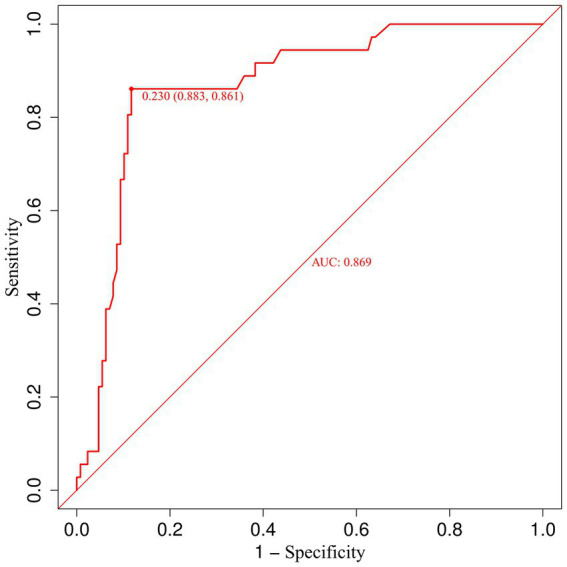
Receiver operating characteristic (ROC) curve for urinary β-2 microglobulin in predicting CMBs.

To further elucidate the relationship between urinary β-2 microglobulin levels and the occurrence of CMBs, we performed a receiver operating characteristic (ROC) curve analysis. This analysis aimed to determine the optimal cut-off value for urinary β-2 microglobulin in predicting CMBs and to assess its diagnostic performance. Figure 7 illustrates the ROC curve for urinary β-2 microglobulin as a predictor of CMBs. The area under the curve (AUC) was 0.87, indicating good discriminative ability. The optimal cut-off value was determined to be 0.23 mg/L, which yielded a sensitivity of 0.88 and a specificity of 0.86. These results suggest that urinary β-2 microglobulin levels above 0.23 mg/L are associated with an increased risk of CMBs, with high sensitivity and specificity.

#### 3.6.3 Subgroup analysis of CMBs risk factors in diabetic patients

Subgroup analysis revealed significant differences in CMBs prevalence between diabetic (37.1%, 13/35) and non-diabetic patients (17.8%, 23/129) (*χ*^2^ = 5.89, *p* = 0.01), as shown in Table 7. Hypertension was significantly associated with CMBs in both diabetic (69.20% vs. 40.90%, *p* = 0.01, OR = 3.25, 95% CI: 1.07–9.88) and non-diabetic patients (82.60% vs. 15.10%, *p* < 0.001, OR = 26.72, 95% CI: 8.29–86.14). Urinary β-2 microglobulin levels were higher in CMBs groups for both diabetic (1.80 ± 1.42 vs. 0.81 ± 0.68 mg/L, *p* = 0.410) and non-diabetic patients (1.60 ± 1.70 vs. 0.66 ± 0.98 mg/L, *p* = 0.716), although not statistically significant. Similarly, CyC levels were elevated in CMBs groups for diabetic (1.27 ± 0.36 vs. 1.03 ± 0.40 mg/L, *p* = 0.58) and non-diabetic patients (1.36 ± 0.60 vs. 0.98 ± 0.30 mg/L, *p* = 0.585). BUN levels were also higher in CMBs groups for both diabetic (7.53 ± 1.19 vs. 5.42 ± 1.30 mmol/L, *p* = 0.117) and non-diabetic patients (6.73 ± 1.80 vs. 5.38 ± 1.41 mmol/L, *p* = 0.90). Two-way ANOVA revealed no significant interaction between diabetes status and CMBs presence for urinary β-2 microglobulin, CyC, or BUN (all *p* > 0.05), suggesting that the relationship between these markers and CMBs is consistent across diabetic and non-diabetic populations.

**Table 7:** Patient demographics and baseline characteristics.

Characteristic	CMBs, *N* = 36			Non-CMBs, *N* = 128		
	Diabetes (No), *N* = 23^a^	Diabetes (Yes), *N* = 13^a^	*p*-value^b^	Diabetes (No), *N* = 106^a^	Diabetes (Yes), *N* = 22^a^	*p*-value^b^
Hypertension			0.422			0.014
Yes	19 (82.6%)	9 (69.2%)		16 (15.1%)	9 (40.9%)	
No	4 (17.4%)	4 (30.8%)		90 (84.9%)	13 (59.1%)	
Urinary β-2 microglobulin (mg/L)	1.60 ± 1.70	1.80 ± 1.42	0.716	0.66 ± 0.98	0.81 ± 0.68	0.410
CyC (mg/L)	1.36 ± 0.60	1.27 ± 0.36	0.585	0.98 ± 0.30	1.03 ± 0.40	0.597
BUN (mmol/L)	6.73 ± 1.80	7.53 ± 1.19	0.117	5.38 ± 1.41	5.42 ± 1.30	0.901

a *n* (%); mean ± SD.

b Fisher’s exact test; Welch two sample *t*-test.

#### 3.6.4 Construct a comprehensive renal function scoring system by integrating multiple renal function indicators

**Figure 8:**
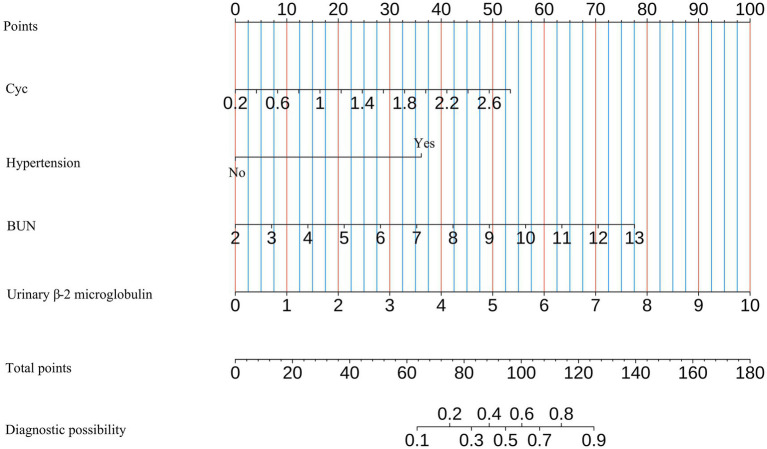
Nomogram for predicting CMBs risk based on renal function indicators and hypertension.

We developed an integrated nomogram (Figure 8) to assess the risk of CMBs based on multiple renal function indicators and associated risk factors. This nomogram incorporates four key variables: CyC, hypertension status, BUN, and urinary β-2 microglobulin. CyC ranges from 0.20 to 2.60 mg/L and contributes the maximum of 100 points, emphasizing its significant impact on CMBs risk. Hypertension, as a binary variable, adds approximately 40 points when present, highlighting its crucial role. BUN ranges from 2 to 13 mmol/L, contributing up to about 60 points, while urinary β-2 microglobulin spans from 0 to 10 mg/L, adding up to 90 points, with higher levels corresponding to higher scores for both variables. The total points range from 0 to 180, translating to a diagnostic possibility for CMBs from 0.1 to 0.9, with intermediate values of 0.2, 0.3, 0.4, 0.5, 0.6, 0.7, and 0.8. This wide range allows for nuanced risk stratification, potentially aiding clinicians in identifying high-risk individuals. The model suggests that elevated levels of renal markers, particularly CyC and urinary β-2 microglobulin, along with the presence of hypertension, significantly increase CMBs likelihood. For example, a CyC level of 1.40 mg/L contributes about 50 points, while a BUN level of 7 mmol/L adds approximately 30 points. This comprehensive approach, integrating both glomerular and tubular function markers with a key vascular risk factor, provides a valuable tool for CMBs risk assessment.

## 4 Discussion

The present study delves into the intricate relationship between renal function indicators and the risk of CMBs through a logistic regression model. Our findings underscore the significant roles of hypertension, BUN, urinary β-2 microglobulin, and CyC as predictors of CMBs. These results highlight the critical intersection between renal and cerebrovascular health, offering new insights into the pathophysiological mechanisms linking these two vital systems.

Hypertension emerged as the most significant risk factor for CMBs (9), corroborating the extensive body of literature that establishes its role in cerebrovascular pathology (11, 12). Hypertension contributes to the development of small vessel disease through several mechanisms, including endothelial dysfunction, inflammatory responses (13), hypoperfusion, oxidative stress (13, 14), and disruption of the blood-brain barrier (15). These pathological processes collectively facilitate the formation of CMBs. The hemodynamic parallels between the deep perforating arteries in the brain and the renal arterioles suggest a shared vulnerability to hypertension-induced damage (16). Sustained high blood pressure exerts continuous stress on these small vessels, leading to structural and functional impairments (17, 18). Henskens et al. (19) demonstrated that nocturnal hypertension, in particular, significantly elevates the risk of CMBs, likely due to the lack of protective blood pressure dips during sleep. This finding underscores the importance of comprehensive blood pressure management, including nocturnal monitoring, to mitigate the risk of CMBs (19). Additionally, hypertension-induced vascular damage is often accompanied by increased expression of pro-inflammatory cytokines such as tumor necrosis factor-alpha (TNF-α) and markers of oxidative stress, which further exacerbate endothelial dysfunction (20, 21). The chronic inflammatory state and oxidative stress associated with hypertension contribute to the breakdown of the blood–brain barrier, making the brain more susceptible to microbleeds (22–24). Therefore, managing hypertension is crucial for reducing the risk of CMBs and preventing further cerebrovascular complications.

Beyond hypertension, renal function indicators also play a crucial role in CMBs development. Although direct studies linking BUN to CMBs are scarce, BUN is a well-established marker of renal function, inflammation, and endothelial dysfunction, all of which are associated with cerebral small vessel disease (CSVD) (25). Elevated BUN levels can indicate renal impairment (26), which shares common vascular risk factors with the brain, including fluctuating hypertension and endothelial dysfunction. For example, an article suggests that oxidative stress and inflammation play a crucial role in endothelial dysfunction and hypertension related organ damage, which is closely related to endothelial dysfunction caused by elevated BUN levels (27). Previous research has linked BUN to increased risk of CSVD (28), suggesting that high BUN levels may indirectly contribute to the development of CMBs through similar pathological mechanisms involving neurohormonal pathways and the renin-angiotensin-aldosterone system (29). The interaction between hypertension and BUN further amplifies the risk of CMBs. Hypertension can exacerbate renal impairment, leading to elevated BUN levels (19). This renal dysfunction, in turn, contributes to systemic endothelial dysfunction and inflammation (30), creating a vicious cycle that enhances the likelihood of cerebrovascular damage and CMB formation (16, 31). Thus, the combined effect of hypertension and elevated BUN underscores the interconnectedness of renal and cerebrovascular health. These findings highlight the need for integrated management strategies to address both hypertension and renal dysfunction to mitigate the compounded risks of cerebrovascular disease (32).

Urinary β-2 microglobulin is another renal function marker that reflects glomerular filtration and tubular reabsorption capabilities (33). Elevated levels of urinary β-2 microglobulin indicate renal dysfunction, which can be associated with systemic small vessel disease, including in the brain (34). This study identified urinary β-2 microglobulin as a significant predictor of CMBs, highlighting the potential of renal biomarkers to serve as indicators of cerebral vascular health. The interaction between hypertension and urinary β-2 microglobulin in CSVD is particularly noteworthy (35, 36). Hypertension can cause glomerular hyperfiltration and tubular damage, leading to increased levels of urinary β-2 microglobulin (34, 37). This renal impairment can reflect systemic small vessel disease, including in the cerebrovascular system, where hypertension already exerts significant detrimental effects (25, 38). Therefore, the combination of elevated blood pressure and urinary β-2 microglobulin levels indicates a compounded risk for CMBs (34). These insights emphasize the value of urinary β-2 microglobulin as a non-invasive marker for assessing cerebrovascular risk in hypertensive patients, thereby aiding in the early detection and management of potential cerebrovascular complications.

CyC is a cysteine protease inhibitor and a sensitive marker of renal function (39). Elevated CyC levels are associated with impaired endothelial function and have been linked to the presence of CMBs in previous studies (40). A meta-analysis explored the relationship between renal function markers (including CyC) and CSVD. The study emphasizes the link between renal dysfunction (marked by elevated CyC) and increased risk of CMBs, mainly due to endothelial dysfunction (25). Our findings corroborate these studies, demonstrating that CyC is a significant predictor of CMBs. CyC’s role in atherosclerotic processes and its involvement in oxidative stress and inflammation further support its relevance as a biomarker for cerebrovascular health (41). At the same time, some studies have pointed out that CyC is not only a marker of renal function, but also related to the formation of atherosclerosis, mainly through oxidative stress and inflammatory pathways. Higher levels of CyC are associated with extracranial carotid atherosclerosis (42). Although a direct causal relationship between CyC levels and CMB pathology remains to be established, its predictive value in our logistic regression model underscores its importance. The interplay between hypertension and CyC is critical in understanding their combined impact on CMBs. Hypertension can lead to renal impairment, reflected by elevated CyC levels (43). This renal dysfunction contributes to systemic endothelial dysfunction, oxidative stress, and inflammation (44, 45), all of which are key mechanisms in the pathogenesis of CMBs. The combined effect of hypertension and elevated CyC levels suggests a heightened vulnerability to cerebrovascular damage, emphasizing the need for integrated management of blood pressure and renal function to mitigate the risk of CMBs (46).

The interaction between the three renal function biomarkers—BUN, urinary β-2 microglobulin, and CyC—provides a comprehensive picture of renal and cerebrovascular interplay (47, 48). Elevated BUN levels indicate impaired renal function and systemic metabolic stress, contributing to endothelial dysfunction and inflammation (49). This condition is often accompanied by increased urinary β-2 microglobulin, reflecting glomerular and tubular damage (50). Both markers together signify a high burden of renal pathology that can translate into systemic vascular damage, including in the brain (51). CyC, a marker for glomerular filtration, further complements this understanding by providing insights into chronic renal impairment and its systemic effects (52). Elevated CyC levels correlate with increased oxidative stress and inflammation, exacerbating the risk of cerebrovascular damage (53). The combined elevation of these biomarkers suggests a compounded risk mechanism where renal impairment and systemic endothelial dysfunction converge to heighten the risk of CMBs (30, 54). This integrated perspective underscores the importance of monitoring multiple renal function indicators to predict and manage cerebrovascular risks effectively. Therefore, a multifaceted approach to monitoring and managing these biomarkers is crucial for preventing cerebrovascular complications and improving patient outcomes.

This study has several limitations that should be considered when interpreting the results. First, as a retrospective analysis, it cannot establish causality between the identified risk factors and CMBs. The observational nature of the study limits the ability to infer direct cause-and-effect relationships. Second, the study population was limited to a single center in China, which may limit the generalizability of the findings to other populations with different demographic and clinical characteristics. Third, potential confounding factors, such as comorbid conditions and medication use, were not evaluated, which could introduce bias into the analysis. Future prospective studies should address these limitations by including diverse populations and evaluating additional confounding variables to validate and extend these findings. Addressing these limitations will enhance the robustness and applicability of future research findings and contribute to a more comprehensive understanding of the interplay between renal function and cerebrovascular health.

## Data Availability

The original contributions presented in the study are included in the article/supplementary material, further inquiries can be directed to the corresponding author.
